# *Arthrospira platensis* Mutagenesis for Protein and C-Phycocyanin Improvement and Proteomics Approaches

**DOI:** 10.3390/life12060911

**Published:** 2022-06-17

**Authors:** Wanida Pan-utai, Siriluck Iamtham, Sittiruk Roytrakul, Sarn Settachaimongkon, Ladda Sangduean Wattanasiritham, Sumitra Boonbumrung, Juta Mookdasanit, Sayamon Sithtisarn

**Affiliations:** 1 Department of Applied Microbiology, Institute of Food Research and Product Development, Kasetsart University, Chatuchak, Bangkok 10900, Thailand; supersayamon@hotmail.com; 2 Department of Science, Faculty of Liberal Arts and Science, Kasetsart University, Kamphaeng Saen, Nakhon Pathom 73140, Thailand; faassli@ku.ac.th; 3 Center for Agricultural Biotechnology, Kasetsart University, Kamphaeng Saen Campus, Nakhon Pathom 73140, Thailand; 4 Center of Excellence on Agricultural Biotechnology: (AG-BIO/PERDO-CHE), Bangkok 10900, Thailand; 5 Functional Ingredients and Food Innovation Research Group, National Center for Genetic Engineering and Biotechnology, 113 Thailand Science Park, Phahonyothin Rd., Pathum Thani 12120, Thailand; sittiruk@biotec.or.th; 6 Department of Food Technology, Faculty of Science, Chulalongkorn University, Pathumwan, Bangkok 10330, Thailand; sarn.s@chula.ac.th; 7 Department of Food Chemistry and Physics, Institute of Food Research and Product Development, Kasetsart University, Chatuchak, Bangkok 10900, Thailand; ifrlds@ku.ac.th (L.S.W.); ifrstb@ku.ac.th (S.B.); 8 Department of Fishery Products, Faculty of Fisheries, Kasetsart University, Chatuchak, Bangkok 10900, Thailand; juta.m@ku.th

**Keywords:** *Arthrospira*, C-phycocyanin, mutagenesis, protein, proteomics

## Abstract

*Arthrospira (Spirulina) platensis* is known for its use as a food supplement, with reported therapeutic properties including antiviral, anti-inflammatory and antioxidant activity. *Arthrospira* is also an excellent source of proteins and C-phycocyanin. The latter is a light-harvesting pigment-protein complex in cyanobacteria, located on the outer surface of the thylakoid membrane and comprising 40 to 60% of the total soluble protein in cells. Random mutagenesis is a useful tool as a non-genetically modified mutation method that has been widely used to generate mutants of different microorganisms. Exposure of microalgae or cyanobacteria to chemical stimuli affects their growth and many biological processes. Chemicals influence several proteins, including those involved in carbohydrate and energy metabolisms, photosynthesis and stress-related proteins (oxidative stress-reactive oxygen species (ROS) scavenging enzymes). Signal transduction pathways and ion transportation mechanisms are also impacted by chemical treatment, with changes causing the production of numerous biomolecules and stimulation of defence responses. This study compared the protein contents of *A. platensis* control and after mutagenesis using diethyl sulphate (DES) under various treatment concentrations for effective mutation of *A. platensis.* Results identified 1152 peptides using proteomics approaches. The proteins were classified into 23 functional categories. Random mutagenesis of *A. platensis* by DES was found to be highly effective for C-phycocyanin and protein production.

## 1. Introduction

Microalgae comprise various groups of photosynthetic aquatic microorganisms that grow in a wide range of habitats, pH values and temperatures. They have simple growth requirements with effective and efficient use of light, carbon dioxide (CO_2_) and other inorganic nutrients [1]. Recently, microalgae have gained considerable attention due to their high growth rate and as a source of valuable compounds such as proteins, lipids, carbohydrates, carotenoids and phycobiliproteins that can accumulate in large quantities. These compounds are used as bioactive food additives, pharmaceuticals, cosmetics and biofuels as renewable energy sources. Culture conditions and species play significant roles in biomolecular accumulation [2]. The market value for microalgae is predicted to be USD 6.5 billion, with food applications accounting for 39%, docosahexaenoic acid production 23% and aquaculture 11% [3]. Microalgae are also an excellent source of natural colourants, with increasing popularity as an alternative to synthetic colourants because they are non-toxic and non-carcinogenic [4]. Industrially significant microalgae are both prokaryotes and eukaryotes [5]. In cyanobacteria, rhodophyta, cryptomonads and cyanell phycobiliproteins are light-harvesting pigment–protein complexes [6] composed of 40–60% of the total soluble protein in cells and found on the outer surface of the thylakoid membrane [7]. Phycobiliproteins are classified into three classes based on their protein structure and absorption spectra as C-phycocyanin (*C*-*PC*), allophycocyanin (APC) and phycoerythrin (PE) [8,9,10]. The primary group of phycobiliproteins is *C*-*PC*, which is commercially generated in photoautotrophic open raceway pond cultivation of the *Arthrospira* *platensis* cyanobacterium [2,11]. The well-known *Arthrospira* (*Spirulina*) *platensis* is a rich source of protein [12] with a well-balanced profile containing all the essential amino acids. Proteins and amino acids from *Arthrospira* are recommended for human consumption by the FAO and WHO [13]. 

*Arthrospira* (commercially known as *Spirulina*) is a multicellular photosynthetic filamentous spiral-shaped blue–green prokaryotic microalga [14]. *Arthrospira* lacks cellulose in the cell membrane, is larger than other microalgal species and is readily digested and absorbed by the human body [15]. *A. platensis* is a rich source of macro and micronutrients including proteins, pigments, lipids, vitamins and minerals. Therefore, it can be used as a functional food with pharmaceutical, medical and cosmetic applications, and also as an antioxidant pigment. Multiple health benefits from *Arthrospira* include antioxidant, antiviral, anti-inflammatory and anticancer activities [16], with favourable benefits against malnutrition, obesity, diabetes and anaemia with no negative side effects [2,11,12,15]. Phycobiliproteins (PBPs), particularly C-phycocyanin, are blue water-soluble and brightly coloured as the principal protein components of *Arthrospira*, with significant health benefits [17]. The market price of phycobiliproteins varies from USD 12 (per 100 mg for food or cosmetic application) to USD 1500 (per 1 mg for reagent or analytical application), depending on purity and pigment grade [18,19]. 

Improved performance of microorganisms is commonly conducted by genetic engineering. However, instability of the transformants and low capacity of foreign gene expression are key barriers limiting progress in enhancing the phenotypes of *Arthrospira* [20]. Random mutagenesis as a non-GM mutagenesis method is a powerful tool that has produced mutant proteins with different properties from wild-type molecules without human bias. Mutagenesis is simple and affordable, requiring little understanding of an organism’s genetic information, metabolic processes and genetic control. It also involves minimal technological modifications and the strains produced are not subject to rigorous legal issues [21]. Various microalgae have been randomly mutated using both chemical and physical mutagenesis to enhance their growth rates and metabolite yield (lipid) and also increase their toxic compound tolerance against environmental stress [22]. Several proteins have been successfully mutated by random mutagenesis methods [20,23]. Chemical mutagenesis involves the use of chemicals that generate mistakes in base pairing, purine deamination, transitions, transversions and frameshift mutations via combination with DNA [24]. Alkylating agents are the most used chemical mutagenic agents, including diethyl sulphate (DES), ethyl methane sulphonate (EMS), methyl methane sulfonate (MMS) and N-methyl-N-nitro-N-nitrosoguanidine (NTG) [24,25]. *Arthrospira* has been subjected to a variety of mutation procedures. Isolated mutants of *A. platensis* cells showed threefold higher tolerance to metronidazole and DCMU (3,4-dichlorophenyl-1,1-dimethylurea) after chemical mutation via nitro-N-nitrosoguanidine (NTG) treatment compared to a wild-type strain, although there was no improvement in *A. platensis* growth with the same mutagen NTG [20,26]. 5-Fluorotryptophan, ß-2-thienyl-alanine, ethionine, p-fluprophenlalanine and azetidine-2-carboxylic acid resistance were recorded in *A. platensis* mutants. A few mutants overproduced related amino acids such as valine or proline, showing greater mutation frequency [27,28]. All chemical mutagenetic approaches must consider the safety of the operator and the environment [20,26]. Among mutagens, DES almost completely alkylated nitrogen sites in DNA bases, with guanine and adenine being the most affected [29]. Therefore, *A. platensis* requires a mutagenetic technique that is efficient, effective, safe and ecologically benign.

Global proteome analyses in response to several stresses, including chemical, are well documented. Proteins are important components of living organisms that play various roles in metabolic pathways [30]. Many recent studies focused on protein quantity, identification of proteins and protein activity. Proteomics is an essential tool to understand biological systems by providing information on protein levels, activity and functions as the next step in the study of overall biological processes [31]. A large variety of algae are characterised in terms of genomics and transcriptomics, while proteomics lacks data information. The eukaryote microalga *Chlamydomonas reinhardtii* has been studied as the first proteomics analysis of algal mitochondria and was used as a model organism [32]. Algae have grown in relevance in the field of energy, particularly in the manufacture of algae-based biofuels, where proteomics investigations have discovered several proteins as well as microalgal lipid accumulation [33,34]. Proteomic algal analysis includes fractionating complicated protein–peptide mixtures using multidimensional protein/peptide separation. This simplifies peptides for LC-MS/MS and allows the acquisition of MS/MS spectra for lower-abundance peptides [35,36]. Proteins and peptides of *Synechocystis* sp. PCC 6803 were compared and 776 proteins were identified [35]. Treatment of *A. platensis* PCC 8005 found 30 proteins with significant differential regulations under light/dark growth transition [36]. Proteomics provides more informative data than genomic studies because an organism’s genome is constant in each species, whereas proteomes differ from cell to cell and throughout time. Thus, the objective of this study was to develop *A. platensis* under diethyl sulphate (DES) mutagenesis treatment for protein and *C*-*PC* enhancement. Protein quantification and identification of control and mutant cells were also evaluated to improve system understanding via proteomic approaches and *C*-*PC* related gene expression confirmation.

## 2. Materials and Methods

### 2.1. Microalgae Preparation

The cyanobacterium *Arthrospira platensis* IFRPD 1182 was obtained from the Institute of Food Research and Product Development, Kasetsart University, Thailand. The starter culture was maintained and prepared in Zarrouk medium [37]. *A. platensis* was cultured and incubated in chamber equipment [38] with temperature controlled at 30 °C. Light intensity was controlled at photon flux density of 162 µmol m^−2^ s^−1^ using fluorescent standard 18 Watt daylight lamps with a 16/8 h light and dark cycle. Flow rate was 2% (*v*/*v*) CO_2_ mixed with air at 0.67 vvm continuous bubbling through a PTFE membrane filter. *A. platensis* was grown for 7 days or until reaching log phase for use in the mutagenesis treatment experiments.

### 2.2. Mutagenesis Treatment

*A. platensis* was prepared by centrifugation (CF-10, Daihan Scientific, Indonesia) at 10,900× *g* for 10 min and the cell pellets were then sonicated for 30 s. The cells were resuspended in diethyl sulphate (DES) to induce random mutagenesis at various concentrations of 0 (control), 0.02, 0.04, 0.06, 0.08, 0.1, 0.2, 0.4, 0.6, 0.8 and 1 M. Treated cells were collected at 10 min intervals up to 60 min and 10% sodium thiosulphate was added to inactivate the mutagen. The collected cells were centrifuged at 10,900× *g* for 10 min and washed twice to remove the DES mutagen. The treated cells were grown in Zarrouk medium and incubated for expression of mutant cells under controlled conditions of temperature 30 °C, light/dark cycle 16/8 h and light intensity with fluorescent standard 18 Watt daylight lamps at photon flux density of 162 µmol m^−2^ s^−1^. All experiments were conducted in triplicate. After 14 days, the mutant *A. platensis* cells were collected by centrifugation at 10,900× *g* for 10 min and stored at −20 °C until required for analysis.

### 2.3. Protein Determination

Protein concentrations were determined following the dye-binding Lowry protein assay [39] using BSA as a standard. The sample or BSA at 5 µL was added to 200 µL of solution A (CTC, 20% Na_2_CO_3_, 0.8 N NaOH and 5% SDS) and incubated for 30 min at room temperature. Then, a 50 µL solution of B (Folin–Ciocalteu reagent at 20% *v*/*v*) was added and incubated in the dark for 30 min at room temperature. The mixture of the sample solution was determined for absorbance at 750 nm. All measurements were conducted in triplicate, with mean values used to represent protein concentration of the experiments.

### 2.4. C-Phycocyanin Determination

Optical density of the extracted samples was measured at 652, 615 and 652 nm using a Spectrophotometer (M965+, Microplate Reader, Metertech, Taiwan). C-phycocyanin (*C*-*PC*) concentration was calculated using equation [11].

(1) $$C-PC\;\left(\mathrm{mg}\;{\mathrm{mL}}^{-1}\right)=\frac{OD_{615}-0.474OD_{652}}{5.34}$$

### 2.5. Proteomic Approaches

#### 2.5.1. In-Solution Protein Digestion

The screening of *A. platensis* mutants was analysed using proteomic techniques. Five micrograms of proteins were decreased with dithiothreitol (DTT) at 10 mM in ammonium bicarbonate (NH_4_HCO_3_) at 10 mM with the temperature controlled at 37 °C for 1 h. The reduced cysteine residues were alkylated by adding iodoacetamide (IAA) in 10 mM ammonium bicarbonate to a final concentration of 20 mM. Samples were incubated at room temperature for 1 h in the dark. Protease trypsin was added to a 1:20 (enzyme/protein) ratio in solution, with digestion taking place overnight at 37 °C. The final digestion solution was incubated at 40 °C. The samples were dried and kept at −80 °C until required for determination by LC-MS/MS. Peptide samples from the digestion were resolved in 15 µL of 0.1% formic acid in LC/MS water grade. Transfer of solution in each well was made to low protein-binding microtubes before centrifuging at 10,900× *g* for 10 min. The clear solution was placed in a vial for Nano LC-MS/MS analysis.

#### 2.5.2. Nano-Liquid Chromatography and Mass Spectrophotometry (Nano LC-MS/MS)

The tryptic peptides were resuspended in 0.1% formic acid following the previous preparation. Triplicate samples were injected into an ion trap mass spectrometer (HCT Ultra Ion Trap, Bruker Daltonics, Germany) linked to a nano-LC system (Ultimate 3000 LC System, Thermo Fisher Scientific, Waltham, MA, USA). The peptide mixture was fractionated using a reverse-phase high-performance liquid chromatography column (Acclaim PepMap^TM^ 100 Å, 75 µm × 5 cm, Thermo Fisher Scientific, Leicestershire, UK and PepSwift Monolithic Trap Column 200 µm × 5 cm Thermo Fisher Scientific, Leicestershire, UK). The mobile phases consisted of buffer A (0.1% formic acid in H_2_O) and buffer B (0.1% formic acid in 80% acetonitrile) as eluting peptides. The elution with linear gradient was run as follows: 4–70% of solvent B at 0–20 min (the time point of retention time), followed by 90% of solvent B at 20–25 min to remove all peptides in the column and 96% solvent A for 15 min for column re-equilibration. Finally, mass spectra of gradient eluted peptides were examined using a MS1 precursor scan (*m*/*z* 400–1500) in Data-Dependent Acquisition (DDA) mode. The five most abundant multiple charged precursor ions were selected for MS2 fragmentation from a MS2 scan (*m*/*z* 200–2800).

#### 2.5.3. Protein Quantitation and Identification

The quantitation of protein was performed using DeCyder MS Differential Analysis software (DeCyderMS, GE Healthcare) [40,41]. The PepDetect module was used to automate peptide identification and charge state assignments, with quantification based on the peptide ion signal intensities in MS mode using acquired LC-MS raw data. Mascot software (Matrix Science, London, UK) was used to search a database for MS/MS data from DeCyderMS. [42]. For protein identification, data were compared to the NCBI database. Based on peptide signal intensities, the average abundance ratio of peptides from the control and stress conditions of phosphopeptides was calculated using the Pepdetect module for automated peptide identification and charge state assignment. Database interrogation included enzyme (trypsin), variable modifications (carbamidomethyl, oxidation of methionine residues), mass values (monoisotopic), protein mass (unrestricted), peptide mass tolerance (1.2 Da), fragment mass tolerance (±0.6 Da), peptide charge state (1+, 2+ and 3+) and max missed cleavages (1). Proteins were identified containing at least one peptide with an individual MASCOT score corresponding to *p* < 0.05.

### 2.6. Confirmation of Mutants

#### 2.6.1. RNA Isolation

Total RNA was isolated from individual *A. platensis* samples. Briefly, cells were grown in Zarrouk medium until they reached the exponential growth phase. The samples were then centrifuged for 30 min at 10,900× *g*. One millilitre of TRI reagent was then added and mixed vigorously. The mixture was added with 200 μL of saturated chloroform, vortexed and incubated for 3 min at room temperature. After centrifugation at 15,000× *g* for 10 min, the upper aqueous phase was transferred to a fresh tube and added with 500 μL of isopropyl alcohol. The mixture was incubated overnight at −20 °C and then centrifuged for 15 min at 15,000× *g*. The layer of supernatant was discarded, and the pellet was washed with 1 mL of 75% ethanol and then dissolved with diethyl pyrocarbonate-treated water.

#### 2.6.2. Reverse Transcription Polymerase Chain Reaction

DNA-free total RNA was extracted from *A. platensis* using a GenUPTM total RNA kit (Biotechrabbit^TM^, Germany) according to the manufacturer’s instructions. Subsequently, cDNA was reverse transcribed from 2 μg total RNA and 1 μg oligo dT according to the manufacturer’s protocol with 10 μL SuPrimeScript RT-PCR Premix (2X) (GeNet Bio, Korea). Taq-DNA polymerase was used to amplify the generated first strand of cDNA using oligo-dT primer. The template for PCR was amplified by gene-specific primers. PCR was started with a 10 min denaturation at 94 °C followed by denaturation at 94 °C for 30 s to 1 min, annealing at 55–63 °C for 1–2 min and elongation at 72 °C for 30 s to 1 min in the presence of 0.2 mM dNTPs, 10 mM forward and reverse primers in Taq DNA polymerase buffer and 0.25 units of Taq DNA polymerase.

#### 2.6.3. Quantitative RT-PCR Analysis

Quantitative RT-PCR analysis was determined for differentially expressed genes among the control and treated samples. The quantitative real-time reverse transcription-PCR technique was used to differentiate the expressed proteins between DES *A. platensis* mutants and the control using an ExicyclerTM 96 Real-Time PCR Instrument (Bioneer, Korea). The reaction mixture consisted of 1 μL of cDNA, 0.4 μL of forward and reverse primers, 5 μL of 5x Hot Firepol Evagreen qPCR Master Mix (Solis BioDyne) and 3.2 μL of nuclease-free water in a total volume of 10 μL. Cycling qPCR parameters for 40 cycles were denaturation for 30 s at 95 °C, annealing for 30 s at 60°C and elongation for 30 s at 72 °C. Finally, the result of qPCR (Ct) was normalised with Ct of 16S RNA per sample. Relative transcript levels of gene expression were calculated using the 2^−ΔΔCT^ method [43]. Student’s t-test was performed to compare resistant and sensitive groups, with significance reported at *p* < 0.05. Measurements of gene expression for all RNA extractions were obtained in triplicate.

## 3. Results and Discussion

*Arthrospira platensis* cells were treated with diethyl sulphate (DES) to initiate chemical mutagenesis among variable concentration and duration conditions to induce high protein and C-phycocyanin accumulation. Mutant cells were selected, and peptides were identified using mass spectrophotometry.

### 3.1. Screening of A. platensis Mutagenesis

Various induced mutagenesis methods are available, and these play an important role in increasing genetic variability. Microalgal enhancement is emerging as a potential strategy for industries looking to expand production of high-value products. In general, industries exploit random mutagenesis and spontaneous mutation, followed by adaptive screening, to develop improved strains with desired properties [44]. One of the most effective techniques to study proteins, genes, molecular pathways and cell developmental events is chemically induced mutagenesis [45]. Selection of efficient chemical mutagens and their treatment concentrations is important for successful mutagenesis to improve the characteristics of cells and create variable genetic and biochemical mechanisms. Random mutagenesis is an effective method for developing desirable, sustainable microalgal characteristics and is also cost effective for industrial use. Chemical mutagenesis has been widely employed to develop microalgal mutants with high biomass productivity, high lipid/pigment content and resistance to abiotic stress. Mutagenesis is also a simple, cost-effective and unbiased approach that does not require prior understanding of microalgae genetics or metabolic pathways [46]. In this study, we explored the possibilities of inducing alterations in protein levels to enhance protein and phycocyanin levels using diethyl sulphate (DES) in *A. platensis* cyanobacterium. 

DES treatments of *A. platensis* IFRPD 1182 were performed at various concentrations and duration times using three datasets for high protein and C-phycocyanin accumulation. Overall soluble protein and *C*-*PC* contents of DES treatments are shown in Figures S1 and S2, respectively. Protein and C-phycocyanin contents fluctuated due to random mutagenesis that did not show trends of significant values (protein and *C*-*PC*). Results indicated protein and *C*-*PC* contents ranging from 298–1160 mg/g dry weight (DW) and 0.01–0.06 mg/g protein, respectively.

Omics methodologies provide a deeper knowledge of the fundamental biological development processes of algae strains [44]. Genomics and transcriptomics elucidate details about cell genetic complexity and expression pattern, whereas proteomics provides information about the proteins that sustain the cell for structural, organisational and metabolic potential. Creation of active proteins involves numerous levels of control of cell proteome profiles under different conditions [47]. A proteomic approach was used to determine whether DES treatment impacted protein level and *C*-*PC* concentration in *A. platensis*. Results showed that DES dosage at 0–1 M induced mutagenesis in cells and initiated cell-response mechanisms. Total proteins were extracted from the DES-treated and control conditions to identify protein and *C*-*PC* contents. Differentially expressed protein profiles of the DES treatments were analysed using the Lowry method. Maximum values for both protein and *C*-*PC* were recorded after 10 min of DES treatment, with highest concentrations found in twelve and eight samples, respectively, after 60 min. Seven samples with the lowest protein content were found under DES treatment after 60 min. Highest protein and *C*-*PC* and lowest protein samples were collected for proteomic analysis using LC-MS/MS (Table 1). 

Proteins are primary targets of mutagenesis due to their chemical treatment characteristics and abundance in cells. Chemical treatment causes protein damage through direct oxidation or protein degradation, leading to loss of functional activities and causing reversible and irreversible modifications such as protein–protein cross linking, glycation, nitration and carbonylation. These changes result in lack of function, fragmentation, unfolding/misfolding, protein aggregation and degradation due to structural, functional and stability alterations. Proteins are biological activity effectors. Here, proteomics was applied to determine the target proteins specifically altered by chemical treatment. The samples were screened and further analysed for protein expression profiles to identify the function of proteins in DES-treated cells compared to the control cells by LC-MS/MS (Table 1). The proteins were classified into three groups: high protein (H protein—HP), low protein (L protein—LP) and high phycocyanin (*C*-*PC*: HCPC).

### 3.2. Identification of Differentially Expressed Proteins

Treatment with DES has been reported to result in alteration of protein expression, i.e., induction and up- or downregulation of sets of proteins. In this study, comparative proteome analysis of the control and DES-treated samples was conducted using LC-MS/MS to identify potential proteins and/or pathways regulated by specific proteins that responded to production of high levels of protein and C-phycocyanin.

Mutagenesis is one of the most widely studied methods for increasing biomass and lipid content in microalgae. Random mutagenesis can be achieved through chemical mutagens, including ethyl methanesulphonate (EMS), *N*-methyl-N′-nitro-N-nitrosoguanidine (MNNG), *N*-methyl-N-nitrosourea and diethyl sulphate (DES) [21,24,48]. Base pairing mistakes, purine deamination, transitions, transversions and frameshift mutations are all caused by chemicals [21]. DES is a monofunctional alkylating chemical that has been demonstrated in a variety of organisms to cause mutations, chromosomal abnormalities and other genetic changes [48]. Moreover, chemicals affect proteins involved in carbohydrate and energy metabolisms, photosynthesis and stress-related proteins (oxidative stress–reactive oxygen species (ROS) scavenging enzymes) [49]. Signal transduction and ion transport mechanisms also alter under chemical treatment, involving changes in signal transduction pathways that then induce numerous biomolecular and defence responses. Proteins also play a vital role in stress response and adaptation. Mutagenesis has been shown to improve microalgae. Hyperproduction of astaxanthin from *Phaffia rhodozyma* mutant microalgae was achieved using UV exposure and ethyl methanosulphonate (EMS) or *N*-methyl-N′-nitro-N-nitrosoguanidine (MNNG) treatment [50], while lipid enhancement from *Chlamydomonas reinhardti* succeeded in using random mutagenesis via EMS [51,52]. Proteomics has emerged as a more effective tool for deciphering the complexities of biological systems. Proteomics is used to study protein expression and interactions combined with genomics and transcriptomics. The major goal of proteomics is to examine post-translational modifications, as well as to determine the varied amounts of proteins in submultiprotein complexes and their locations in various types of cells and tissues. Proteomics can also help us to understand metabolic processes. Protein expression and differences are primarily influenced by various environmental stimuli, as well as the metabolic pathway in which they are involved, using proteomics analysis [33,53]. Protein digestion by the endoprotease trypsin is the most widely used approach for investigating the complexity of protein samples in large-scale proteomics investigations. Analysis of digested peptides using mass spectrometry is also an important method to clarify protein profiles. Mass spectrometry, as an instrument developed in the last two decades, is used to measure and identify individual molecules while tandem mass spectrometry (MS/MS) involves multiple steps of mass spectrometry.

**Figure 1:**
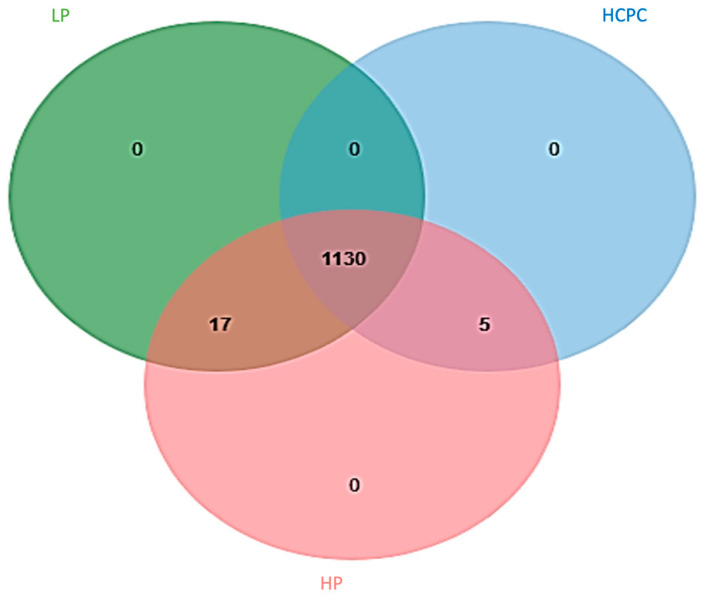
Venn diagram identifying 1152 proteins as high protein (HP), low protein (LP) and high phycocyanin (HCPC) matched with the *Spirulina* database.

Here, differentially expressed proteins were identified by LC-MS/MS (*p*-values < 0.05). For identification of the control and DES-treated samples, MASCOT Daemon 2.3.0 generated a monoisotopic peak list file (MASCOT generic format, MGF) from raw data collected from LC-MS/MS. The MGF files were compared to the other green microalgae (NCBI) database on an in-house server. Trypsin was used as the enzyme restriction, with a maximum of three missed cleavages permitted. Cysteine carbamidomethylation was designated as a permanent modification, whereas methionine, histidine and tryptophan oxidation were designated as variable modifications. A total of 1152 proteins were matched to the MASCOT database (NCBI *Spirulina* database). These proteins were separated into three groups as high protein (HP), low protein (LP) and high phycocyanin (HCPC). Five proteins were found in both the high protein (HP) and high phycocyanin (HCPC) groups, as shown in Figure 1.

**Figure 2:**
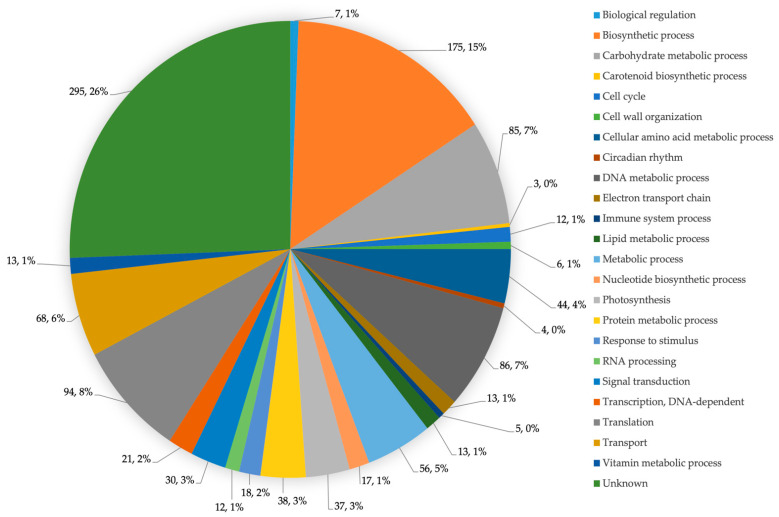
Functional categories of identified proteins from *A. platensis* mutagenesis screening.

All proteins can be classified into 23 functional categories comprising biological regulation, the biosynthetic process, the carbohydrate metabolic process, the carotenoid biosynthetic process, cell cycle, cell wall organisation, the cellular amino acid metabolic process, the circadian rhythm, the DNA metabolic process, electron transport chain, the immune system process, the lipid metabolic process, the metabolic process, the nucleotide biosynthetic process, photosynthesis, the protein metabolic process, response to stimulus, RNA processing, signal transduction, transcription, translation, transport, the vitamin metabolic process and unknown. Functional distributions and protein percentages based on their functions in the proteome are shown in Figure 2. 

**Figure 3:**
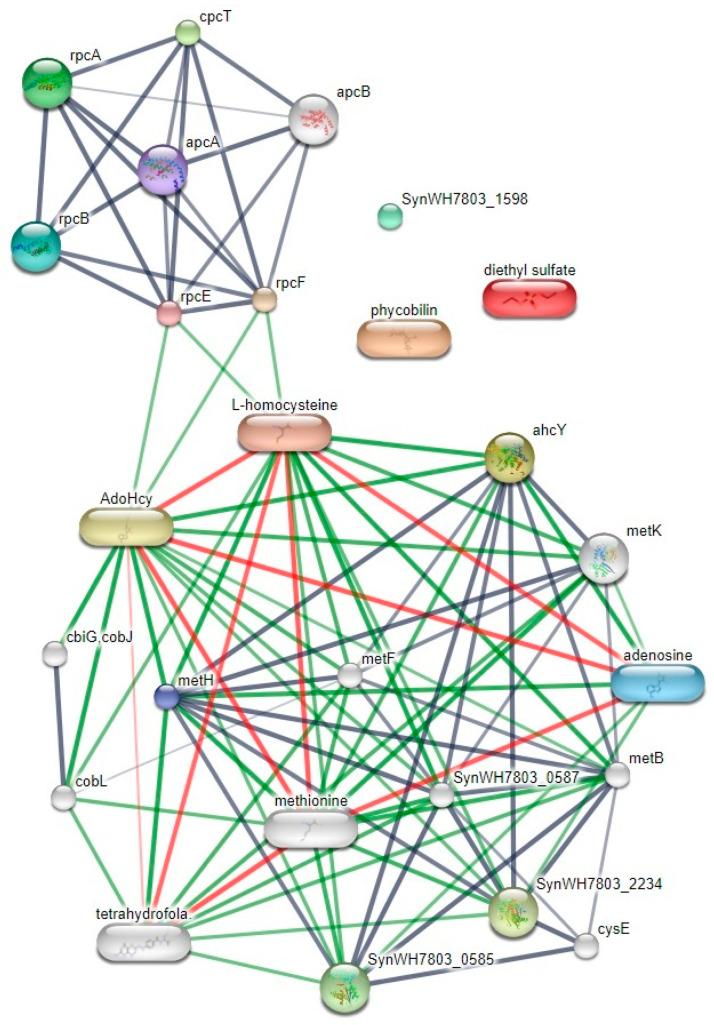
Interactive map of five proteins and their functional partners by STITCH 5.0 analysis.

The Stitch database (version 5.0; http://stitch.embl.de/) (accessed on 21 September 2021) analysis showed protein–protein and protein–chemical interactions of five proteins, including adenosyl homocysteinase, prephenate dehydratase (PDT), beta-lactamase family protein, ABC1 domain-containing protein and thiamine–phosphate synthase (TP synthase) that gave high expression levels in both groups of high protein and high phycocyanin. Interactive networks of candidate proteins and their functional partners are shown in Figure 3. The interactive map indicated no correlation between these five proteins and DES molecules and phycobilin. However, ahcY (S-adenosyl-L-homocysteine hydrolase) correlated with molecular and protein expression as amino acid synthesis, while ahcY (S-adenosyl-L-homocysteine hydrolase) correlated with protein expression for *C*-*PC* production as L-homocysteine, rpcF, rpcE, rpcB, apcA, apcB, rpcA and cpcT.

**Figure 4:**
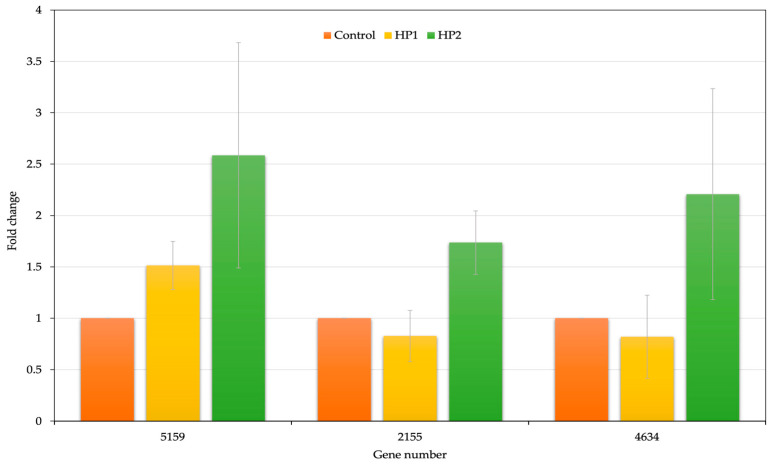
Expression profiles of three genes involved in phycocyanin DES-treated synthesis of control and mutant cells. Fold change (log_2_) in relative expression was determined by qRT-PCR after normalisation to the 16S rRNA gene at three time points relative to the control using three individual replicates (Gene numbers 5159—phycobilisome protein; 2155—phycobilisome linker polypeptide and 4634—phycocyanin, alpha subunit).

Confirmation of *A. platensis* mutants from DES random mutagenesis treatment was repeated under control, HP1 and HP2 conditions. Comparisons between transcript levels of the three genes involved in phycocyanin biosynthesis of the DES-treated and control samples are shown in Figure 4. Differentially expressed genes (DEGs) were identified from the DES-treated and control samples. Upregulation of three genes was observed for the HP2 condition. All three genes were observed upregulated in HP2 condition with more than 1.5-fold differential expression. All three genes (ORF 5159, ORF 2155 and ORF 4634) were involved in energy metabolism, including photosynthesis and the respiratory electron transport pathway evolved by cyanobacteria to dissipate excess energy and limit cellular damage. A more comprehensive understanding of the complexity of these systems and their roles in enabling cyanobacteria to survive under varying environmental conditions has recently advanced knowledge in this area [54,55]. Moreover, the two genes ORF 4634 and 2155 were related to energy production and conversion found in *Arthrospira*. Differential gene expressions (DEGs) showed increased response under DES compared with the control.

## 4. Conclusions

The ability of *Arthrospira platensis* cyanobacterium to induce accumulation of proteins and C-phycocyanin in cells was proved by DES treatment. Proteins were quantified, with 1152 peptides identified. The proteins were classified into 23 functional categories. Protein and C-phycocyanin production were correlated using a proteomic analysis tool. An *A. platensis* mutant was confirmed using the expression profile. Random mutagenesis of *A. platensis* by DES was highly effective for C-phycocyanin and protein production.

## Figures and Tables

**Table 1:** Screening of DES-treated *A. platensis* for protein quantitation and identification.

Mutant Name	Diethyl Sulphate			Protein Conc.	*C*-*PC* Conc.
	Concentration (M)	Time (min)	Number of Datasets	(mg/g DW)	(mg/g Protein)
HP1	0	10	3	1159.474	0.051
HP2	0.8	10	1	1140.934	0.047
HP3	0.06	20	3	850.519	0.013
HP4	0.6	20	3	846.150	0.019
HP6	0.8	20	1	861.553	0.037
HP7	0.04	30	2	681.932	0.010
HP8	0	40	3	776.859	0.012
HP9	0.2	40	1	782.994	0.017
HP10	0	50	3	892.505	0.018
HP11	0.6	60	1	588.925	0.014
HP12	0.6	60	2	600.163	0.014
LP1	0	10	1	505.735	0.060
LP2	0.04	10	2	502.440	0.108
LP3	0.02	20	1	574.638	0.022
LP4	0.08	30	2	297.856	0.018
LP5	0.1	40	3	390.843	0.038
LP6	0.02	50	1	309.871	0.029
LP7	0.08	60	2	533.735	0.045
HCPC1	0.04	10	3	975.342	0.049
HCPC2	0.8	10	1	1140.934	0.047
HCPC3	0.08	20	1	649.668	0.056
HCPC4	0.02	30	3	508.691	0.032
HCPC5	0.4	40	2	577.667	0.027
HCPC6	0	50	3	892.505	0.018
HCPC7	0.06	50	3	476.228	0.036
HCPC8	0.1	60	3	668.382	0.025

## Data Availability

Not Applicable.

## Supplementary Materials

The following supporting information can be downloaded at: https://www.mdpi.com/article/10.3390/life12060911/s1, Figure S1: Protein content from *A. platensis* mutagenesis treated by DES at various conditions; Figure S2: C-phycocyanin content from *A. platensis* mutagenesis treated by DES at various conditions.

## References

[1] Khan M.I., Shin J.H., Kim J.D. (2018). The promising future of microalgae: Current status, challenges, and optimization of a sustainable and renewable industry for biofuels, feed, and other products. Microb. Cell Factories.

[2] Hsieh-Lo M., Castillo G., Ochoa-Becerra M.A., Mojica L. (2019). Phycocyanin and phycoerythrin: Strategies to improve production yield and chemical stability. Algal Res..

[3] Mobin S., Alam F. (2017). Some Promising Microalgal Species for Commercial Applications: A review. Energy Procedia.

[4] Kuddus M., Singh P., Thomas G., Al-Hazimi A. (2013). Recent developments in production and biotechnological applications of C-phycocyanin. BioMed Res. Int..

[5] Kratzer R., Murkovic M. (2021). Food Ingredients and Nutraceuticals from Microalgae: Main Product Classes and Biotechnological Production. Foods.

[6] Aftari R.V., Rezaei K., Mortazavi A., Bandani A.R. (2015). The optimized concentration and purity of *Spirulina platensis* C-phycocyanin: A comparative study on microwave-assisted and ultrasound-assisted extraction methods. J. Food Process. Preserv..

[7] Padyana A.K., Bhat V.B., Madyastha K., Rajsashankar K., Ramakumar S. (2001). Crystal structure of a light-harvesting protein C-phycocyanin from *Spirulina platensis*. Biochem. Biophys. Res. Commun..

[8] Sonani R.R., Rastogi R.P., Patel R., Madamwar D. (2016). Recent advances in production, purification and applications of phycobiliproteins. World J. Biol. Chem..

[9] Khan Z., Wan Maznah W.O., Faradina Merican M.S.M., Convey P., Najimudin N., Alias S.A. (2019). A comparative study of phycobilliprotein production in two strains of *Pseudanabaena* isolated from Arctic and tropical regions in relation to different light wavelengths and photoperiods. Algal Res..

[10] García A.B., Longo E., Bermejo R. (2021). The application of a phycocyanin extract obtained from *Arthrospira platensis* as a blue natural colorant in beverages. J. Appl. Phycol..

[11] Pan-utai W., Iamtham S. (2019). Extraction, purification and antioxidant activity of phycobiliprotein from *Arthrospira platensis*. Process Biochem..

[12] Lucakova S., Branyikova I., Hayes M. (2022). Microalgal Proteins and Bioactives for Food, Feed, and Other Applications. Appl. Sci..

[13] Lupatini A.L., Colla L.M., Canan C., Colla E. (2017). Potential application of microalga Spirulina platensis as a protein source. J. Sci. Food Agric..

[14] Hachicha R., Elleuch F., Ben Hlima H., Dubessay P., de Baynast H., Delattre C., Pierre G., Hachicha R., Abdelkafi S., Michaud P. (2022). Biomolecules from Microalgae and Cyanobacteria: Applications and Market Survey. Appl. Sci..

[15] Manirafasha E., Murwanashyaka T., Ndikubwimana T., Rashid Ahmed N., Liu J., Lu Y., Zeng X., Ling X., Jing K. (2018). Enhancement of cell growth and phycocyanin production in *Arthrospira* (*Spirulina*) *platensis* by metabolic stress and nitrate fed-batch. Bioresour. Technol..

[16] Dalla Costa V., Filippini R., Zusso M., Caniato R., Piovan A. (2022). Monitoring of Spirulina Flakes and Powders from Italian Companies. Molecules.

[17] Li Y., Aiello G., Bollati C., Bartolomei M., Arnoldi A., Lammi C. (2020). Phycobiliproteins from *Arthrospira Platensis* (*Spirulina*): A New Source of Peptides with Dipeptidyl Peptidase-IV Inhibitory Activity. Nutrients.

[18] Ojit S., Indrama T., Oinam G., Avijeet S., Subhalaxmi S.A., Silvia C., Indira D., Romi K., Minerva S., Thadoi D. (2015). The response of phycobiliproteins to light qualities in *Anabaena circinalis*. J. Appl. Biol. Biotechnol..

[19] Park J., Lee H., Dinh T.B., Choi S., De Saeger J., Depuydt S., Brown M.T., Han T. (2022). Commercial Potential of the Cyanobacterium *Arthrospira maxima*: Physiological and Biochemical Traits and the Purification of Phycocyanin. Biology.

[20] Fang M., Jin L., Zhang C., Tan Y., Jiang P., Ge N., Heping L., Xing X. (2013). Rapid Mutation of *Spirulina platensis* by a New Mutagenesis System of Atmospheric and Room Temperature Plasmas (ARTP) and Generation of a Mutant Library with Diverse Phenotypes. PLoS ONE.

[21] Carino J.D.G., Vital P.G. (2022). Characterization of isolated UV-C-irradiated mutants of microalga *Chlorella vulgaris* for future biofuel application. Environ. Dev. Sustain..

[22] Arora N., Yen H.-W., Philippidis G.P. (2020). Harnessing the power of mutagenesis and adaptive laboratory evolution for high lipid production by Oleaginous microalgae and yeasts. Sustainability.

[23] Tachioka M., Sugimoto N., Nakamura A., Sunagawa N., Ishida T., Uchiyama T., Igarashi K., Samejima M. (2016). Development of simple random mutagenesis protocol for the protein expression system in *Pichia pastoris*. Biotechnol. Biofuels.

[24] Saxena S. (2015). Strategies of strain improvement of industrial microbes. Applied Microbiology.

[25] Schüler L., Greque de Morais E., Trovão M., Machado A., Carvalho B., Carneiro M., Maia I., Soares M., Duarte P., Barros A. (2020). Isolation and Characterization of Novel Chlorella Vulgaris Mutants with Low Chlorophyll and Improved Protein Contents for Food Applications. Front. Bioeng. Biotechnol..

[26] Singh D., Singh N. (1997). Isolation and characterization of a metronidazole tolerant mutant of the cyanobacterium *Spirulina platensis* exhibiting multiple stress tolerance. World J. Microbiol. Biotechnol..

[27] Riccardi G., De Rossi E., Milano A., De Felice M. (1988). Mutants of Spirulina platensis resistant to valine inhibition. FEMS Microbiol. Lett..

[28] Riccardi G., Sora S., Ciferri O. (1981). Production of amino acids by analog-resistant mutants of the cyanobacterium *Spirulina platensis*. J. Bacteriol..

[29] Hoffmann G.R., Crowley D.J., Theophiles P.J. (2002). Comparative potencies of induction of point mutations and genetic duplications by the methylating agents methylazoxymethanol and dimethyl sulfate in bacteria. Mutagenesis.

[30] Kosová K., Vítámvás P., Urban M.O., Prášil I.T., Renaut J. (2018). Plant Abiotic Stress Proteomics: The Major Factors Determining Alterations in Cellular Proteome. Front. Plant Sci..

[31] Rose J.K., Bashir S., Giovannoni J.J., Jahn M.M., Saravanan R.S. (2004). Tackling the plant proteome: Practical approaches, hurdles and experimental tools. Plant J..

[32] van Lis R., Atteia A., Mendoza-Hernández G., González-Halphen D. (2003). Identification of novel mitochondrial protein components of Chlamydomonas reinhardtii. A proteomic approach. Plant Physiol..

[33] Anand V., Singh P.K., Banerjee C., Shukla P. (2017). Proteomic approaches in microalgae: Perspectives and applications. 3 Biotech.

[34] Moellering E.R., Benning C. (2010). RNA interference silencing of a major lipid droplet protein affects lipid droplet size in *Chlamydomonas reinhardtii*. Eukaryot. Cell.

[35] Gan C.S., Reardon K.F., Wright P.C. (2005). Comparison of protein and peptide prefractionation methods for the shotgun proteomic analysis of *Synechocystis* sp. PCC 6803. Proteomics.

[36] Matallana-Surget S., Derock J., Leroy B., Badri H., Deschoenmaeker F., Wattiez R. (2014). Proteome-Wide Analysis and Diel Proteomic Profiling of the Cyanobacterium *Arthrospira platensis* PCC 8005. PLoS ONE.

[37] Zarrouk C. (1996). Contribution à l’étude d’une Cyanophycée. Influence de Divers Facteurs Physiques et Chimiques sur la Croissanceet la Photosynthèse de *Spirulina maxima*. Ph.D. Thesis.

[38] Pan-utai W., Poopat N., Parakulsuksatid P. (2020). Photoautotrophic Cultivation of Arthrospira maxima for Protein Accumulation under Minimum Nutrient Availability. Appl. Food Biotechnol..

[39] Lowry O.H., Rosebrough N.J., Farr A.L., Randall R.J. (1951). Protein measurement with the Folin phenol reagent. J. Biol. Chem..

[40] Johansson C., Samskog J., Sundström L., Wadensten H., Björkesten L., Flensburg J. (2006). Differential expression analysis of *Escherichia coli* proteins using a novel software for relative quantitation of LC-MS/MS data. Proteomics.

[41] Thorsell A., Portelius E., Blennow K., Westman-Brinkmalm A. (2007). Evaluation of sample fractionation using micro-scale liquid-phase isoelectric focusing on mass spectrometric identification and quantitation of proteins in a SILAC experiment. Rapid Commun. Mass Spectrom..

[42] Perkins D.N., Pappin D.J., Creasy D.M., Cottrell J.S. (1999). Probability-based protein identification by searching sequence databases using mass spectrometry data. Electrophoresis.

[43] Livak K.J., Schmittgen T.D. (2001). Analysis of relative gene expression data using real-time quantitative PCR and the 2(-Delta Delta C(T)) Method. Methods.

[44] Sirohi R., Joun J., Choi H., Gaur V.K., Sim S.J. (2021). Algal glycobiotechnology: Omics approaches for strain improvement. Microb. Cell Factories.

[45] Sikora P., Chawade A., Larsson M., Olsson J., Olsson O. (2011). Mutagenesis as a Tool in Plant Genetics, Functional Genomics, and Breeding. Int. J. Plant Genom..

[46] Arora N., Philippidis G.P. (2021). Microalgae strain improvement strategies: Random mutagenesis and adaptive laboratory evolution. Trends Plant Sci..

[47] McLean T.I. (2013). “Eco-omics”: A review of the application of genomics, transcriptomics, and proteomics for the study of the ecology of harmful algae. Microb. Ecol..

[48] Hoffmann G.R. (1980). Genetic effects of dimethyl sulfate, diethyl sulfate, and related compounds. Mutat. Res./Rev. Genet. Toxicol..

[49] El-Nashar Y.I., Asrar A.A. (2016). Phenotypic and biochemical profile changes in calendula (*Calendula officinalis* L.) plants treated with two chemical mutagenesis. Genet. Mol. Res..

[50] Tripathi U., Venkateshwaran G., Sarada R., Ravishankar G.A. (2001). Studies on Haematococcus pluvialis for improved production of astaxanthin by mutagenesis. World J. Microbiol. Biotechnol..

[51] Lee B., Choi G.-G., Choi Y.-E., Sung M., Park M.S., Yang J.-W. (2014). Enhancement of lipid productivity by ethyl methane sulfonate-mediated random mutagenesis and proteomic analysis in *Chlamydomonas reinhardtii*. Korean J. Chem. Eng..

[52] Fu W., Chaiboonchoe A., Khraiwesh B., Nelson D.R., Al-Khairy D., Mystikou A., Alzahmi A., Salehi-Ashtiani K. (2016). Algal Cell Factories: Approaches, Applications, and Potentials. Mar. Drugs.

[53] Gasch A.P., Spellman P.T., Kao C.M., Carmel-Harel O., Eisen M.B., Storz G., Botstein D., Brown P.O. (2000). Genomic expression programs in the response of yeast cells to environmental changes. Mol. Biol. Cell.

[54] Lea-Smith D.J., Bombelli P., Vasudevan R., Howe C.J. (2016). Photosynthetic, respiratory and extracellular electron transport pathways in cyanobacteria. Biochim. Biophys. Acta BBA-Bioenerg..

[55] Huili W., Xiaokai Z., Meili L., Dahlgren R.A., Wei C., Jaiopeng Z., Chengyang X., Chunlei J., Yi X., Xuedong W. (2013). Proteomic Analysis and qRT-PCR Verification of Temperature Response to *Arthrospira* (*Spirulina*) *platensis*. PLoS ONE.

