# Supplementary material for: Arthrospira platensis Mutagenesis for Protein and C-Phycocyanin Improvement and Proteomics Approaches
Source: Life (Basel). 2022 Jun 17;12(6):911. doi: 10.3390/life12060911 (PMC9227609; doi:10.3390/life12060911)
Supplement: Supplementary file 1 [file life-12-00911-s001.zip › life-1755944-supplementary.pdf]

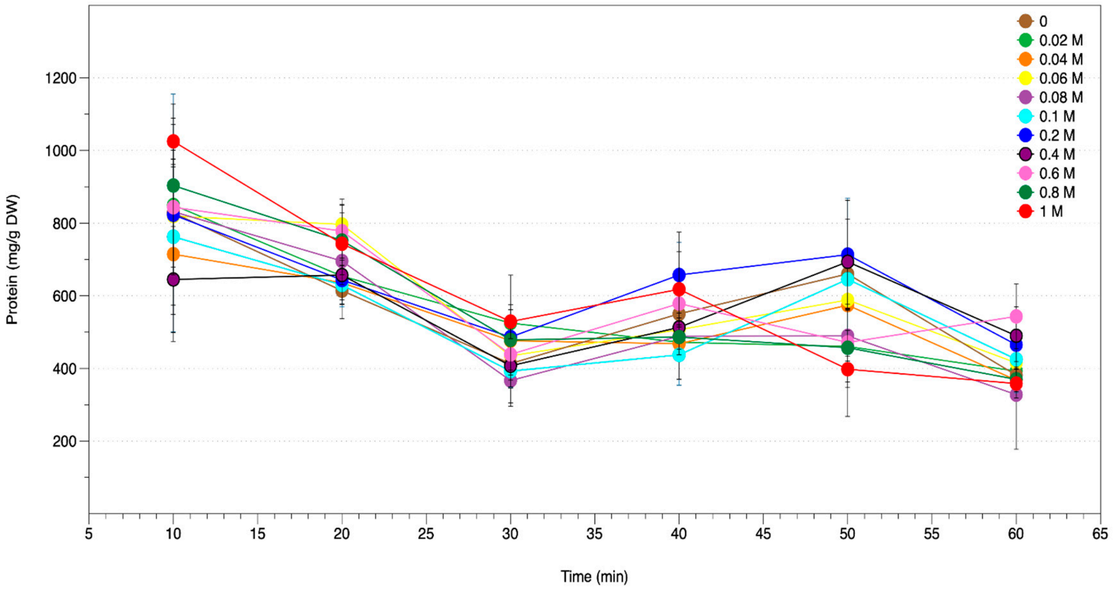

Figure S1. Protein content from *A. platensis* mutagenesis treated by DES at various conditions.

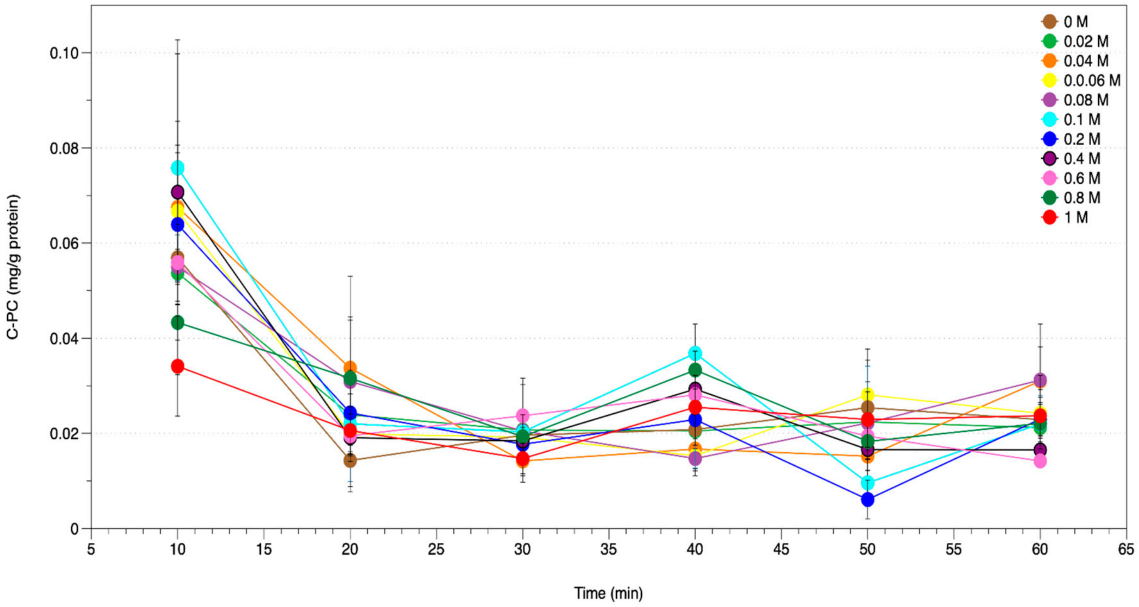

Figure S2. C-phycocyanin content from *A. platensis* mutagenesis treated by DES at various conditions.
